# *PARP10* is highly expressed and associated with inferior outcomes in acute myeloid leukemia

**DOI:** 10.18632/aging.204832

**Published:** 2023-07-27

**Authors:** Ling Wang, Chuang Jiang, Dandan Hu

**Affiliations:** 1 Department of Child Healthcare, Guangzhou Women and Children’s Medical Center, Guangzhou Medical University, Guangzhou, China; 2 Guangzhou Key Laboratory of Child Neurodevelopment, Guangzhou Women and Children’s Medical Center, Guangzhou Medical University, Guangzhou, China; 3 Bioland Laboratory, Guangzhou Regenerative Medicine and Health Guangdong Laboratory, Guangzhou, China; 4 Guangzhou National Laboratory, Guangzhou, China

**Keywords:** *PARP10*, acute myeloid leukemia, prognosis, biomarker

## Abstract

Acute myeloid leukemia is a heterogeneous disease of the hematopoietic system, which possesses a poor prognosis; thus, the identification of novel molecular markers is urgently needed to better define the risk stratification and optimize treatment therapies for this disease. Here, we investigated the roles of the PARP family genes in AML by analyzing their mRNA expression profiles and their association with clinical features using data from TCGA and GSE. Our results showed that *PARP10* was significantly more highly expressed in AML samples than in normal controls, and high expression of *PARP10* was associated with older age (≥60 years, *P* = 0.012), more frequent TP53 mutations (*P* = 0.024), high-risk stratification (*P* < 0.05), and poorer outcomes (*P* < 0.05). Patients with high expression of *PARP10* exhibited significantly poorer overall survival (OS) and event-free survival (EFS) than those with low *PARP10* expressions (OS: median: 0.88 vs. 2.19 years; *P* = 0.001; EFS: median: 0.65 vs. 1.12 years; *P* = 0.041). Multivariate analysis indicated that high expression of *PARP10* was an independent risk factor for poorer OS and EFS in AML patients. Moreover, we found that allo-SCT improved OS for AML patients with high *PARP10* expression but not for patients with low *PARP10* expression, while allo-SCT decreased EFS for patients with low *PARP10* expression. Finally, we confirmed that *PARP10* knockout impaired AML cell proliferation *in vitro*. In summary, our data suggested that *PARP10* is aberrantly expressed in AML, and high expression of *PARP10* might be a biomarker for poor prognosis and also a potential indicator for allo-SCT therapy, which might provide precise treatment indications for physicians.

## INTRODUCTION

Acute myeloid leukemia (AML) is a highly heterogeneous disease characterized by dysplasia and dysdifferentiation of primitive and immature myeloid cells in the bone marrow and peripheral blood cells. Moreover, it represents the most common acute leukemia in adults [1–3]. Risk stratification based on cell morphology and cytogenetics-guided conventional therapies in combination with newly developed molecular targeted regimens and hematopoietic stem cell transplantation (HSCT) have greatly improved the prognosis of AML patients, the current survival rate of AML patients remains poor [4–7]. There is an urgent need for the identification of new biomarkers and the molecular mechanisms involved in leukemogenesis and its progression in order to better define the prognostic subgroups and guide rational disease management of AML.

The poly-ADP-ribose polymerase (PARP) family of genes encompasses 17 members that encode ADP-ribosyl transferases enzymes, which are responsible for the post-translational DNA-damage-dependent modification of nuclear proteins that contribute toward a variety of DNA-repair processes [8–11]. The role of poly (ADP-ribose) polymerases (PARP) in malignancy is well established in BRCA1/2 mutant tumors that are known to be deficient in homologous recombination mechanisms, which leads to their unique susceptibility to PARP1/2 inhibition treatment [12, 13]. In spite of their therapeutic promise in breast and ovarian cancer, the clinical application of PARP inhibitors (PARPis) as an effective treatment has not been widely translated to different cancers, partly because mutations affecting DDR-associated genes are not common in other malignancies, including AML [9, 12–15]. Thus, the dependence on PARPs in other malignancies is not well understood. Intriguingly, there was a recent study by Esposito et al. that demonstrated for the first time a potential utility of PARPi-induced lethality for leukemias driven by AML1-ETO and PML-RARa [15]. Moreover, another study by Molenaar et al. showed that IDH1/2 mutations sensitized AML to PARP inhibition [14], potentiating the possibility of targeting PARPs in AML therapy.

However, the transcriptional expression features and clinical significance of diverse PARP proteins in acute myeloid leukemia (AML) have not been fully established. In this study, we analyzed the association between PARP genes expression and clinical prognostic significance from TCGA and GEO databases to uncover the potential dependence on diverse PARPs in AML and the molecular functions of PARP proteins in AML progression, which may further guide the clinical treatments by targeting PARPs in AML.

## METHODS

### Patients’ data

The RNA expression data, clinical and laboratory parameters data, gene mutation data, and survival data of 173 newly diagnosed AML patients in the TCGA dataset were downloaded from the cBioPortal dataset. The expression differences in PARP family members between AML patients and normal patients were analyzed based on three datasets, i.e., TCGA versus GTEx (UCSC Xena project) [16], GSE15061 [17], and Bloodspot [18].

### Gene differential expression analysis

The gene expression data of AML patients and normal patients downloaded from the GSE15061 dataset [17] was normalized before the differential expression analysis of AML patients versus normal patients was performed using an unpaired *t* test. The gene expression data of the TCGA and GTEx datasets downloaded from the UCSC Xena project were already computed by a standard pipeline; therefore, differential expression analysis was conducted using an unpaired *t* test [16]. Bloodspot included gene expression data, whereby human normal hematopoiesis cells were from GSE42519 [19] and human AML cells were from GSE13159 [20].

### Bioinformatics analysis

*PARP10* co-expression analysis was conducted using cBioPortal based on 173 RNA-seq datasets from TCGA AML patients [21]. KEGG pathway analysis of the significant co-expressed genes was conducted using Enrichr [22]. Gene set enrichment analysis (GSEA) of the *PARP10* high expression versus low expression groups was conducted using GESA software (Broad) [23].

### Cell culture, plasmids, virus package, and cell proliferation assay

Lenti-X 293T cells (Clontech, USA) were cultured in Dulbecco’s Modified Eagle Medium (DMEM, Gibco, USA) with 1 mM sodium pyruvate, 4500 mg/L glucose, and 10% fetal bovine serum (FBS). Cas9-expressing MOLM13 (MOLM13-Cas9) cells were cultured in Roswell Park Memorial Institute (RPMI, USA) 1640 (Thermo Fisher Scientific, USA) with 10% FBS. Cells used in this study were cultured with a medium containing 1/100 Penicillin–Streptomycin (Gibco).

LentiCas9-blast (#52962) and lentiGuide-RFP-blasticidin (#167930) were purchased from Addgene (USA). Two sgRNAs [24] were synthesized, annealed, and inserted into a lentiGuide-RFP-blasticidin plasmid. Lentiviral particles containing sgRNAs were produced by transient transfection of Lenti-X 293T cells using the lipofection method. MOLM13-Cas9 cells were incubated with lentiGuide-RFP-blasticidin lentiviral supernatants for 48 hours, and then, subjected to selection with blasticidin (30 ug/mL) for 3 days. A flow cytometry-based growth competition assay was performed by using lentivirus-infected cells without blasticidin selection. Pooled cells were subjected to flow cytometry to detect the percentage of RFP-positive cells at the indicated days, and the percentage of RFP-positive cells was normalized to day 0. The cell proliferation assay was performed by plating 0.1 × 10^6^ cells per well in a 12 well-plate in triplicate, and monitoring the cell density every two days.

### Statistical analyses

Median expression of *PARP10* was used as threshold to define *PARP10* high or low group in the datasets. The relationship between *PARP10* expression and the clinical and laboratory parameters was analyzed using the Chi-square test. Moreover, the Kaplan-Meier method and log-rank test were used to generate survival curves and to analyze the survival differences between the different groups. Univariate and multivariate analyses were performed using Cox proportional hazards model. All of the statistical analyses were performed using SPSS 24.0 software (SPSS Inc., Chicago, IL, USA), R software, and Prism GraphPad. All tests were two-sided and statistical significance was *P* < 0.05.

## RESULTS

### Differentially expressed *PARP*s in AML versus normal bone marrow cells

**Figure 1:**
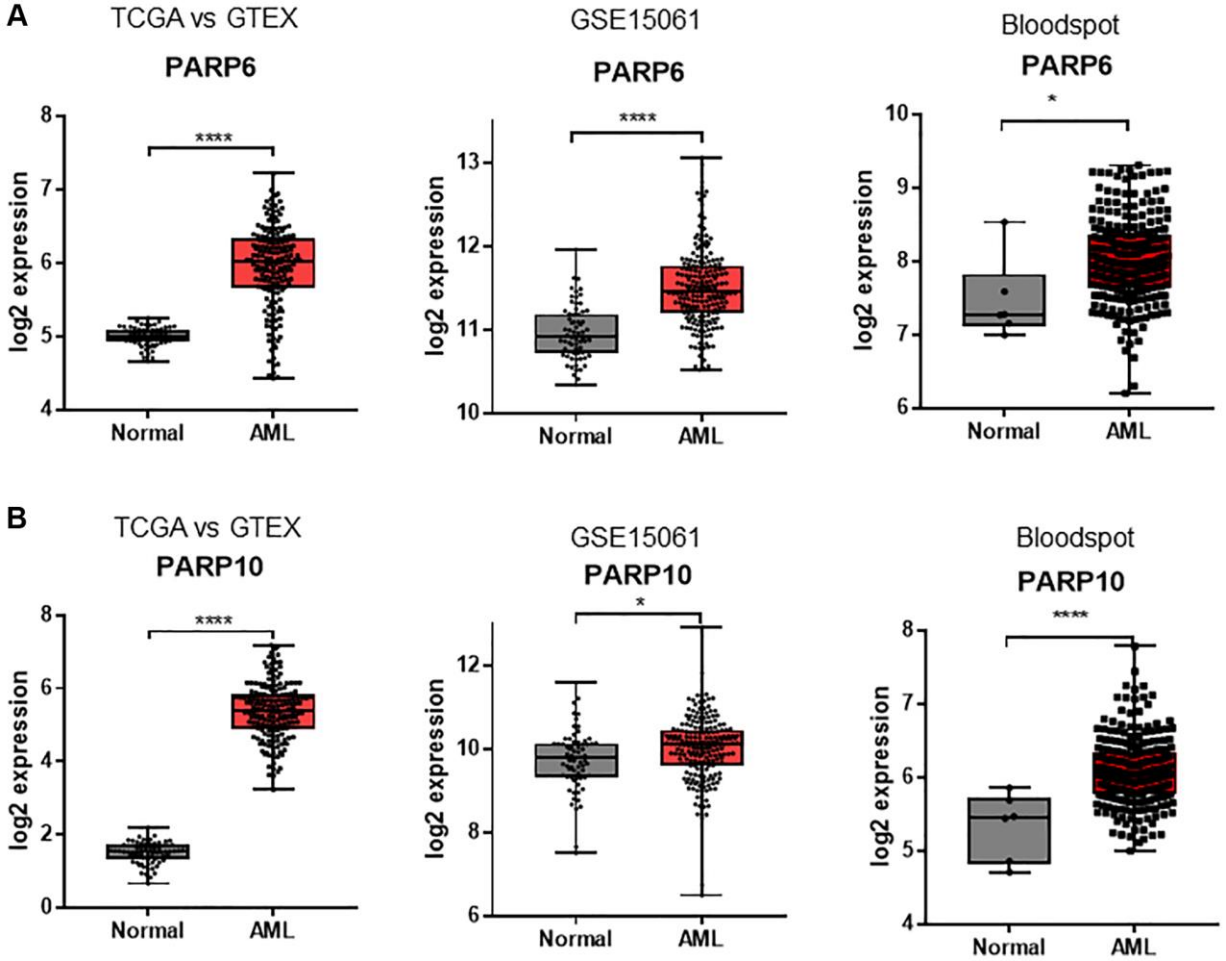
**Expression differences of *PARPs* between AML samples and normal controls in TCGA vs. GTEX, GSE15061, and Bloodspot datasets.** (**A**) *PARP6* and (**B**) *PARP10*. An unpaired *t* test was used to estimate the significant differences in expression. ^*^P < 0.05; ^****^P < 0.001.

To explore the transcriptional expression profiles of *PARP* genes in AML, we first analyzed the mRNA levels of the TCGA versus GTEX databases. The results showed that *PARP1, PARP2, PARP3, PARP4, PARP5b, PARP6, PARP8, PARP9, PARP10, PARP11, PARP12, PARP13, PARP14, PARP15*, and *PARP16* were differentially expressed in AML patients compared to normal bone marrow cells (*P* < 0.05, Figure 1 and Supplementary Figure 1). Next, we conducted a validation analysis of the GEO database (GSE15061) and found that *PARP3, PARP6, PARP10*, and *PARP11* genes exhibited a similar differential expression pattern, whereby these genes showed significantly higher expressions in AML patients compared to normal bone marrow cells, in both databases (Figure 1 and Supplementary Figure 2). The other validation was performed in the Bloodspot dataset, where only *PARP6* and *PARP10* exhibited significantly higher expressions in AML compared to equivalent normal cells (Figure 1 and Supplementary Figure 3). Therefore, *PARP6* and *PARP10* genes might play potential roles in AML progression.

### High *PARP10* expression was associated with poorer survival in AML

**Figure 2:**
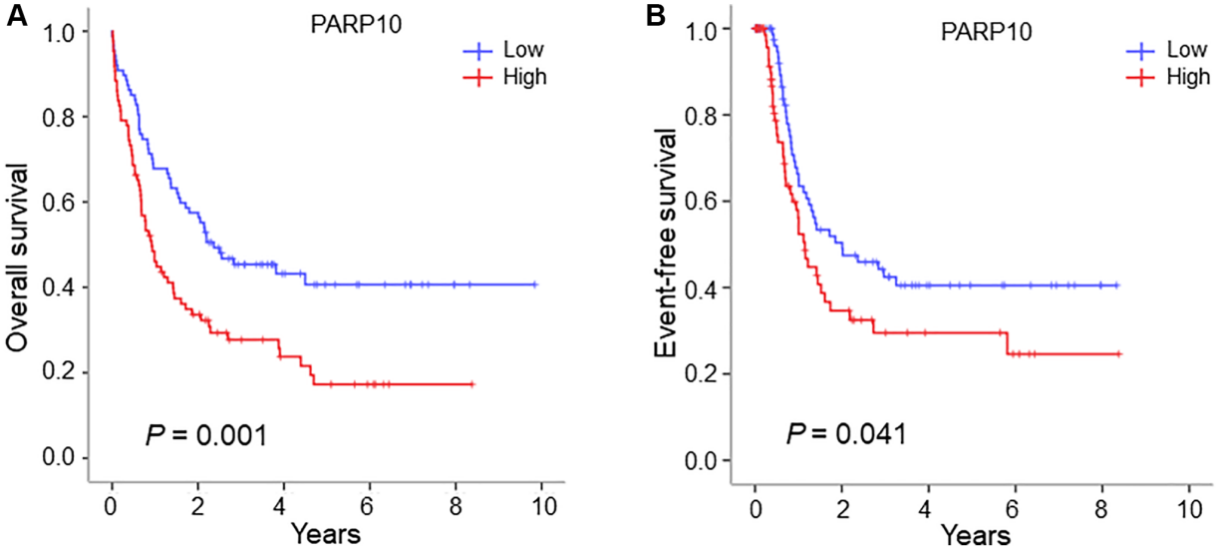
**Survival analysis of AML patients with high expression versus low expression in the PARP10 group.** (**A**) Overall survival difference of AML patients with high *PARP10* expression versus low *PARP10* expression. (**B**) Event-free survival difference of AML patients with high *PARP10* expression versus low *PARP10* expression. Log-rank test was used to generate the survival curves and analyze the survival difference between the high- and low-expression groups.

To better understand the significance of these two genes (*PARP6* and *PARP10*)—in the prognosis of AML patients, we generated Kaplan-Meier survival curves for the overall survival (OS) and event-free survival (EFS) of patients with high and low expression of these genes (cutoff: median expression). Results showed that patients with higher expressions of *PARP10* were significantly associated with poorer OS and EFS compared to patients with lower expressions of *PARP10* (OS: median: 0.88 vs. 2.19 years; *P* = 0.001; EFS: median: 0.65 vs. 1.12 years; *P* = 0.041), while the expression of *PARP6* showed no influence on the prognosis of AML patients (Figure 2 and Supplementary Figure 4). Therefore, we identified *PAPR10* as the only gene among the *PARP* family that was highly expressed and associated with poorer outcomes in AML.

### Association of *PARP10* expression with clinical characteristics from the TCGA database

**Figure 3:**
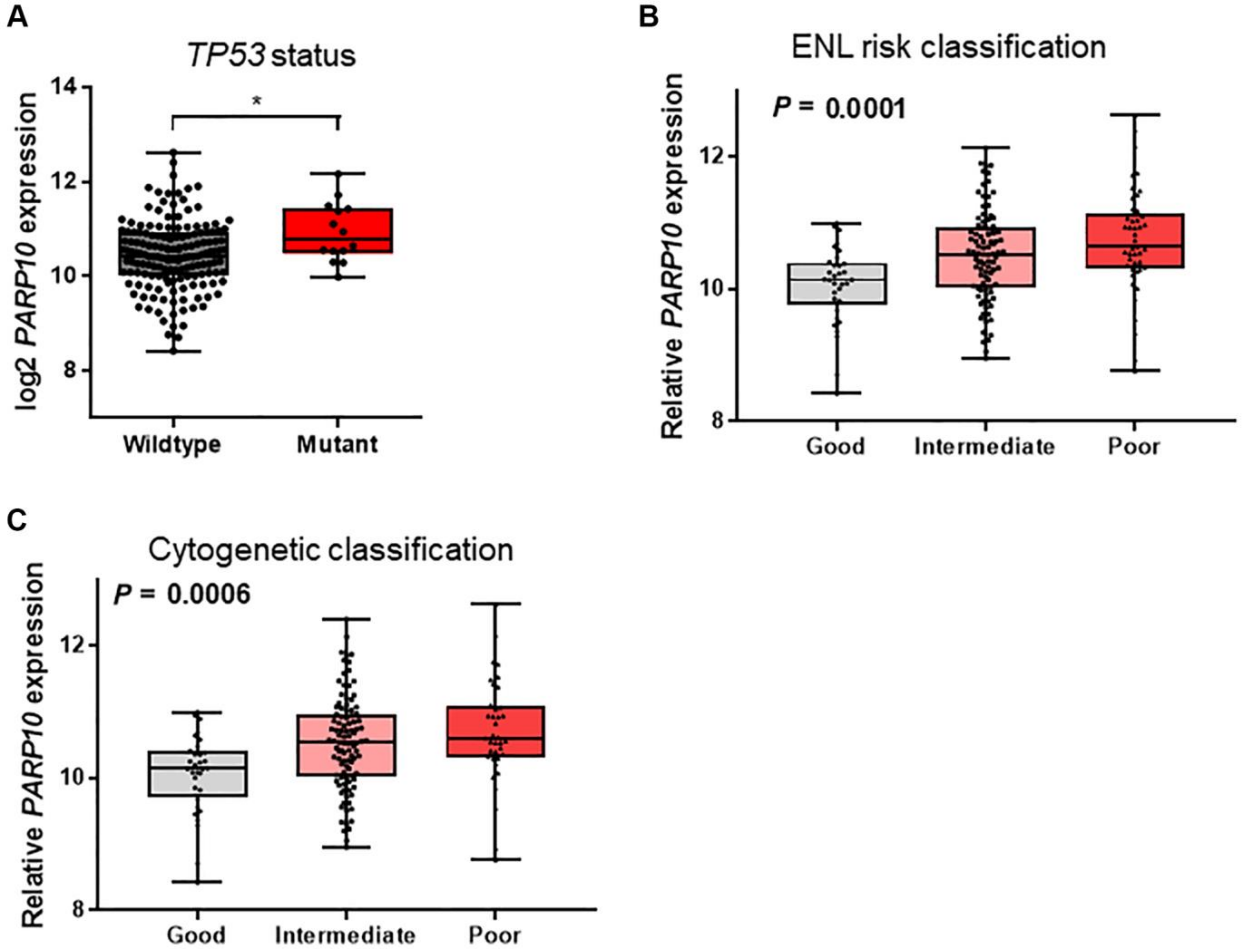
**Association of PARP10 expression with TP53 mutation status and risk classifications in AML patients.** (**A**) *PARP10* expression difference between AML patients with wildtype and mutant *TP53.* Statistical significance was estimated using an unpaired *t* test. (**B**) *PARP10* expression differences among patients with good-risk, intermediate-risk, and poor-risk ENL classifications. (**C**) *PARP10* expression difference among patients with good-risk, intermediate-risk, and poor-risk cytogenetic classifications. Statistical significance was estimated using the Kruskal-Wallis test.

We further analyzed the association between *PARP10* expression and the clinical characteristics in AML patients taken from the TCGA database (Table 1). We found that the patients present in the high expression *PARP10* subgroup were older (≥60 years of age, *P* = 0.012), possessed more frequent TP53 mutations (*P* = 0.024), and had shorter overall (*P* = 0.001) and event-free survivals (*P* = 0.01). Moreover, the patients who possessed the mutant *TP53* showed relatively higher expressions of *PARP10* compared to patients carrying the wildtype *TP53* (*P* < 0.05, Figure 3A). *PARP10* expression was also significantly associated with FAB subtypes (*P* = 0.013), ELN risk classification (*P* = 0.001), and cytogenetic risk classification (*P* < 0.001). According to ELN risk classification, patients with poorer risks exhibited the highest expression of *PARP10*, while patients with good risk exhibited the lowest expression of PARP10 (*P* = 0.001, Figure 3B); cytogenetic risk classification showed a similar pattern (*P* = 0.006, Figure 3C). No significant differences in sex, white blood cells, bone marrow blasts, peripheral blood blasts, and allo-SCT status were observed in the *PARP10* high expression versus low expression groups (*P* > 0.05) (Table 1). Overall, these data indicated that the high expression of *PARP10* was a feature of higher-risk AML, which is more frequently seen in older AML patients, *TP53* mutant patients, and those with high-risk karyotypes, suggesting its clinical significance in predicting poorer clinical outcomes of AML patients.

**Table 1:** Clinical characteristics of AML patients in PARP10 low and high groups from TCGA dataset.

**Clinical parameters**	***PARP10* low**	***PARP10* high**	***P***
**Sex, male/female**	**41/46**	**51/35**	**0.109**
**Age, years (range)**	**56 (18–82)**	**62 (22-88)**	**0.012**
**WBC, ×109/L (range)**	**25.9 (0.4–297.4)**	**14.0 (0.6-223.8)**	**0.656**
**BM, % (range)**	**72.0 (30–100)**	**72.5 (32-99)**	**0.69**
**PB, % (range)**	**44.0 (0–98)**	**35.5 (0-97)**	**0.548**
**Gene mutations**			
* NPM1*, wildtype/mutant	62/25	63/23	0.770
* FLT3*, wildtype/mutant	60/27	64/22	0.426
* CEBPA*, wildtype/mutant	82/5	78/8	0.375
* IDH1/IDH2*, wildtype/mutant	72/15	69/17	0.669
* TP53*, wildtype/mutant	84/3	75/11	0.024
* NRAS/KRAS*, wildtype/mutant	78/9	76/10	0.787
**CRC**			
Favorable	26	6	0
Intermediate	44	48	
Poor	17	29	
**ELN risk stratification**			
Favorable	26	6	0.001
Intermediate	47	54	
Poor	14	23	
**FAB**			
M0	7	9	0.013
M1	16	28	
M2	19	19	
M3	14	2	
M4	22	12	
M5	6	12	
M6	1	1	
M7	1	2	
**Allo-SCT, NO/Yes**	**45/42**	**55/31**	**0.103**
**OS, years**	**2.19 (0–9.84)**	**0.88 (0.01–8.38)**	**0.001**
**EFS, years**	**1.12 (0–8.33)**	**0.65 (0.01–8.38)**	**0.01**

### Univariate and multivariate Cox regression analyses identified *PARP10* as an independent factor of AML prognosis

To evaluate the prognostic value of *PARP10* expression in the presence of other clinical and molecular factors, we included the following dichotomous variables in univariate and multivariate Cox regression analyses: *PARP10* expression levels (high vs. low), age (<60 vs. >60), sex (male vs. female), WBC count (<30 vs. ≥30 × 10^9^/L), eight common gene mutations (*NPM1*, *FLT3*, *CEBPA*, *IDH1/IDH2*, *TP53*, and *NRAS/KRAS*; wildtype vs. mutant), and transplant status (no vs. yes) (Table 2).

Univariate Cox regression analysis showed that higher *PARP10* expression had an adverse effect on both OS (HR = 1.840, 95% CI 1.007–2.171; *P* = 0.001) and EFS (HR = 1.567, 95% CI 1.015–2.420; *P* = 0.043). Lower WBC (<30 × 10^9^/L) and being younger in age (<60 years old) were associated with favorable EFS (HR = 0.485, 95% CI 0.314–0.749; *P* = 0.001) and OS (HR = 0.319, 95% CI 0.219–0.466; *P* < 0.001), respectively. Wildtype FLT3 and TP53 were also associated with a more favorable EFS (HR = 0.585 95% CI 0.368–0.930; *P* = 0.023) and OS (HR = 0.373 95% CI 0.205–0.679; *P* < 0.001), respectively. Furthermore, not having received transplant contributed to an inferior OS (HR = 1.925, 95% CI 1.314–2.821; *P* = 0.001), while the other clinical and molecular factors had no effect on either OS or EFS (*P* > 0.05) (Tables 2 and 3).

**Table 2:** Cox regression analysis of multivariable for overall survival in AML patients.

**Variables**	**OS**			
	**Univariate analysis**		**Multivariate analysis**	
	**Hazard ratio (95% CI)**	***P* value**	**Hazard ratio (95% CI)**	***P* value**
WBC	0.824 (0.569–1.195)	0.308		
Age	0.319 (0.219–0.466)	**0.000**	0.420 (0.278–0.634)	**0.000**
Sex	1.056 (0.731–1.526)	0.77		
*PARP10*	1.840 (1.267–2.673)	**0.001**	1.478 (1.007–2.171)	**0.046**
*NPM1*	0.87 (0.58–1.30)	0.486		
*FLT3*	0.755 (0.504–1.129)	0.171		
*CEBPA*	1.077 (0.545–2.129)	0.931		
*IDH1/IDH2*	1.188 (0.733–1.927)	0.484		
*TP53*	0.244 (0.136–0.437)	**0.000**	0.373 (0.205–0.679)	**0.001**
*NRAS/KRAS*	0.945 (0.53–1.683)	0.846		
Transplant	1.925 (1.314–2.821)	**0.001**	1.455 (0.972–2.179)	0.069

**Table 3:** Cox regression analysis of multivariable for event-free survival in AML patients.

**Variables**	**EFS**			
	**Univariate analysis**		**Multivariate analysis**	
	**Hazard ratio (95% CI)**	***P* value**	**Hazard ratio (95% CI)**	***P* value**
WBC	0.485 (0.314–0.749)	**0.001**	0.477 (0.301–0.755)	**0.002**
Age	0.71 (0.452–1.115)	0.137		
Sex				
*PARP10*	1.567 (1.015–2.420)	**0.043**	1.678 (1.069–2.634)	**0.024**
*NPM1*	0.753 (0.47–1.206)	0.238		
*FLT3*	0.585 (0.368–0.930)	**0.023**	0.597 (0.369–0.966)	**0.036**
*CEBPA*	0.705 (0.339–1.466)	0.349		
*IDH1/IDH2*	1.181(0.674–2.071)	0.561		
*TP53*	0.422 (0.169–1.055)	0.065	0.328 (0.124–0.867)	**0.025**
*NRAS/KRAS*	0.779 (0.402–1.511)	0.460		
Transplant	0.647 (0.414–1.01)	0.055	0.734 (0.467–1.154)	0.180

Multivariate Cox regression analysis indicated that high *PAPR10* expression and *TP53* status were independent risk factors for both OS and EFS, even in the presence of the other covariates (*P* < 0.05, Tables 2 and 3). Age group was also an independent risk factor for OS (*P* < 0.001) after adjusting for the *PARP10* group, *TP53*, and transplant status. The WBC group and *FLT3* status were independent risk factors for EFS after adjusting for the *PARP10* group, *TP53*, and transplant status (*P* < 0.05). Therefore, our Cox regression analyses identified high *PARP10* expression as an independent risk factor for both OS and EFS in AML patients.

### Allo-SCT improved the prognosis of AML patients with high *PARP10* expression but not patients with low *PARP10* expression

**Figure 4:**
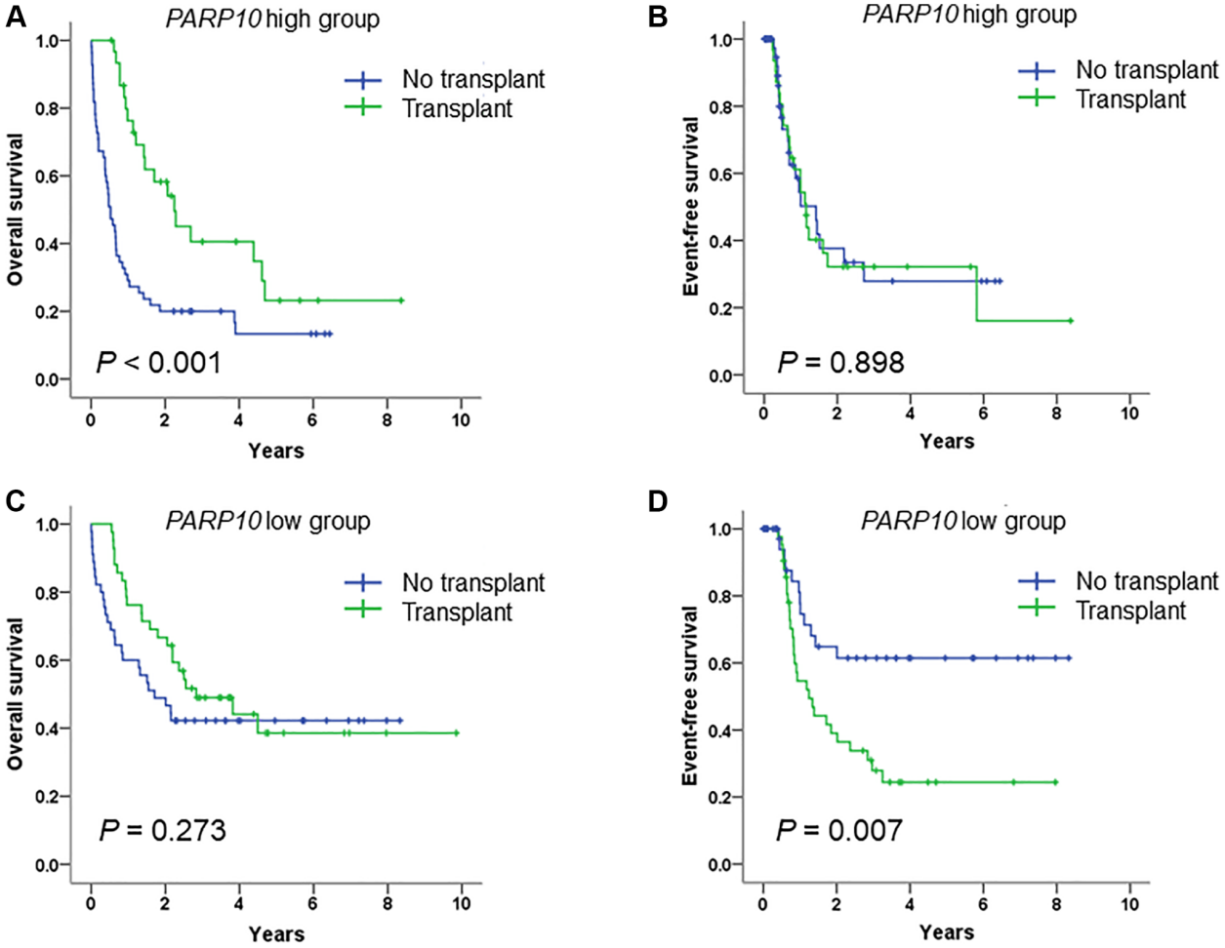
**Survival analysis of AML patients who received intensive chemotherapy or intensive chemotherapy followed by allo-SCT, according to PARP10 expression.** Overall survival rate (**A**) and event-free survival rate (**B**) of AML patients who received intensive chemotherapy versus intensive chemotherapy followed by allo-SCT in the *PARP10* high group. Overall survival rate (**C**) and event-free survival rate (**D**) of AML patients who received intensive chemotherapy versus intensive chemotherapy followed by allo-SCT in the *PARP10* low group.

As allo-SCT has a profound positive influence on the prognosis of AML [25, 26], we further analyzed the influence of transplant status on OS and EFS in *PARP10* high and low AML patients (Figure 4). The results showed that among patients with a high expression of *PARP10*, allo-SCT significantly increased their OS compared to the group without transplants (*P* < 0.001), while there was no difference in EFS between these two groups (*P* = 0.898) (Figure 4A and 4B). Among patients with a low expression of *PARP10*, allo-SCT did not increase their OS compared to the group without transplants (*P* = 0.273), whereas allo-SCT significantly decreased the EFS compared to the group without transplants (*P* = 0.007) (Figure 4C and 4D). These results suggest that allo-SCT therapy can benefit patients with a higher expression of *PARP10* while having an adverse effect on those with a lower expression of *PARP10*. Thus, our results suggest that allo-SCT therapy is highly recommended for patients with higher expressions of *PARP10*, while it is inappropriate for patients with low expressions of *PARP10*.

### Potential molecular mechanism mediated by *PARP10* in AML

**Figure 5:**
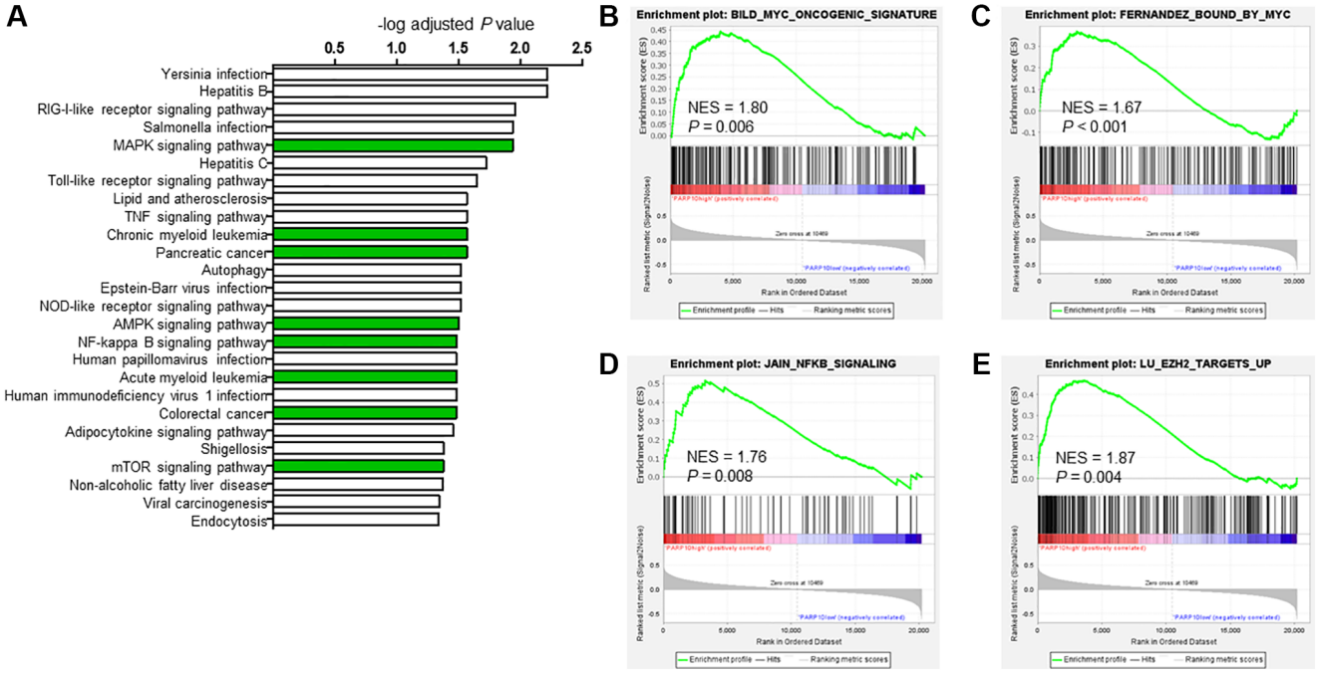
**Potential biological functions of PARP10 in AML.** (**A**) KEGG analysis of *PARP10* positively co-expressed genes (r > 0.5, *P* < 0.05). Significantly enriched pathways (adjust *P* < 0.05) were plotted. And (**B**–**E**) GSEA analysis of AML patients based on *PARP10* expression. NES: normalized enrichment score.

To further explore the mechanism of *PARP10* in AML, we performed co-expression network analysis using RNA sequencing data from the 173 AML patients taken from the cBioPortal database [21] and found 558 positively co-expressed genes (r > 0.5; *P* < 0.05) and 94 negatively co-expressed genes (r < −0.5; *P* < 0.05) (Supplementary Table 1). KEGG pathway analysis revealed that the 558 positively co-expressed genes were significantly enriched in the oncogenic pathways (e.g., MAPK signaling pathway, AMPK signaling pathway, NF-kappa B signaling pathway, mTOR signaling pathway, and pancreatic cancer), and intriguingly, the chronic myeloid leukemia and acute myeloid leukemia pathways were also among the top enriched pathways (adjusted *P* < 0.05), indicating that *PARP10* is highly relevant to AML, potentially through these functional pathways (Figure 5A and Supplementary Table 2). However, KEGG pathway analysis of the 94 negatively co-expressed genes (r < −0.5; *P* < 0.05) did not illustrate any significantly enriched pathways (adjusted *P* > 0.05) (Supplementary Table 3), although this could be due to the limited number of genes.

We next performed gene set enrichment analysis (GSEA) to further explore the involved biological pathways and cofactors of *PARP10* in AML. Median expression of *PARP10* was used as threshold to define *PARP10* high or low group in GSE15061 dataset. For the *PARP10* high expression group, the gene sets were significantly enriched in the *MYC* oncogenic signature (NES = 1.8; *P* = 0.006), *MYC* binding (NES = 1.67; *P* = 0.000), *NFKB* signaling (NES = 1.76; *P* = 0.008), and *EZH2* targets (NES = 1.87; *P* = 0.004) (Figure 5B–5E). STRING protein–protein interaction network analysis also demonstrated interaction of PARP10 and MYC (Supplementary Figure 5). As *MYC, NFKB*, and *EZH2* are key transcriptional factors and epigenetic regulators in tumorigenesis [27–29], our data suggest that *PARP10* could be involved in transcription and epigenomic regulation in AML.

### *PARP10* knockout impaired AML cell proliferation *in vitro*

**Figure 6:**
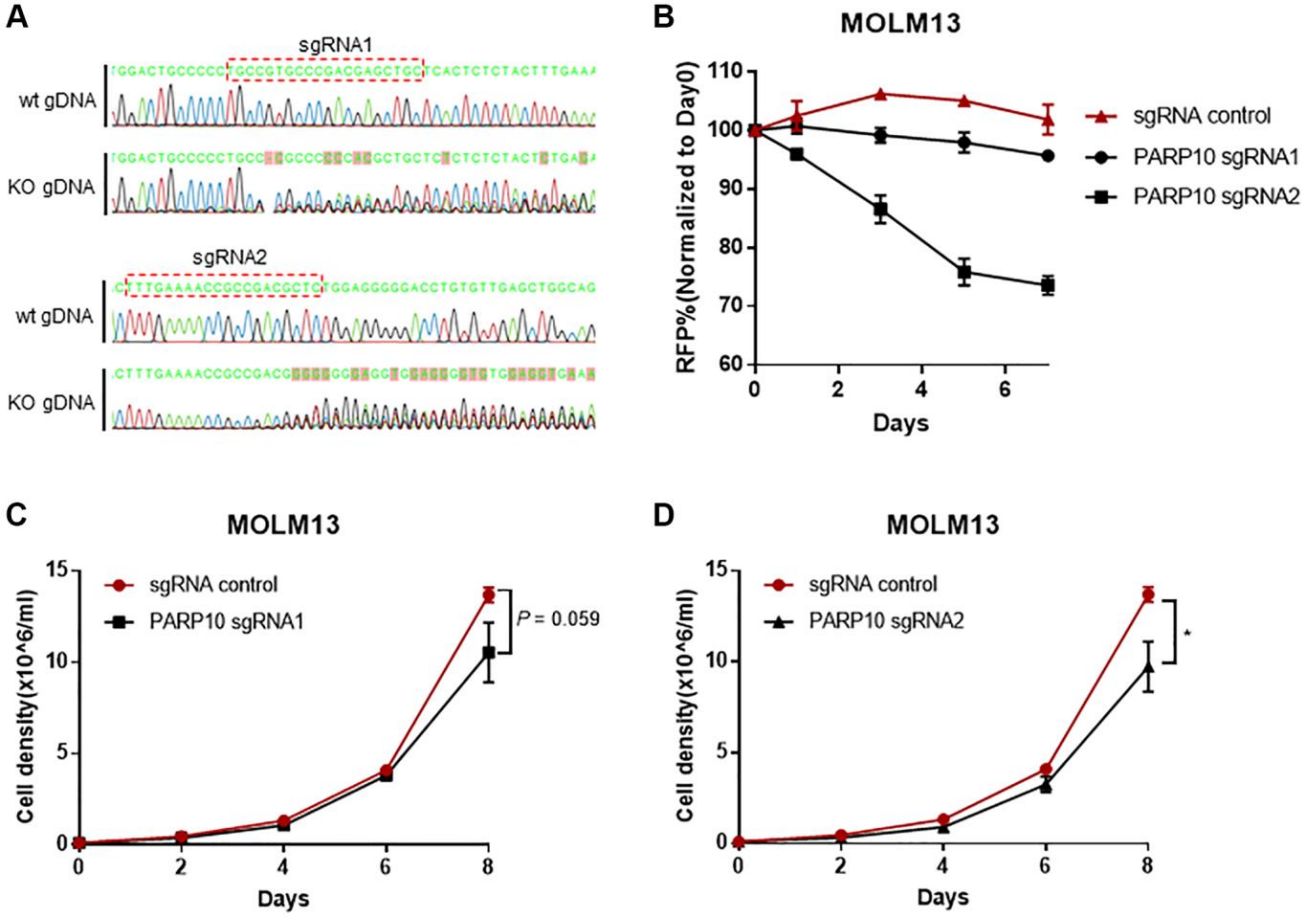
**Loss of *PARP10* impaired proliferation of AML cells.** (**A**) Sanger sequencing showing random insertions and/or deletions introduced by CRISPR/Cas9-mediated *PARP10* editing in MOLM13 cells. (**B**) Flow cytometry-based RFP competition assay showing *PARP10* knockout by two sgRNAs impaired cell proliferation in MOLM13 cells. Proliferation curves of *PARP10* knockout cells introduced by sgRNA1 (**C**) and sgRNA2 (**D**) versus control MOLM13 cells, the error bars represent the SD from triplicates. The asterisk indicates significant statistical differences between the *PARP10* knockout and control cells.

To further gain insight into the function of *PARP10* in AML proliferation, we performed *PARP10* knockout in the MOLM13 cell line using CRISPR/Cas-mediated gene editing. Genotyping of the genomic DNA regions that encompass targeting sites by two guide RNAs [24] successfully validated the random insertions and/or deletions introduced by CRISPR/Cas9-mediated *PARP10* editing in MOLM13 cells (Figure 6A and Supplementary Table 4). In growth competition assays, which tracked the percentage of sgPARP10–RFP cells in MOLM13-Cas9 cells relative to the non-transduced, the RFP negative cells at days 0, 1, 3, 5, and 7 indicated that the expression of sgPARP10 reduced the growth of MOLM13-Cas9 cells compared to the sgEV control cells (Figure 6B). We also established proliferation curves for the control and *PARP10* knockout cells and found *PARP10* knockout, by guide RNA1, impaired the proliferation of MOLM13 cells, although it’s not significant at day 8 (*P* = 0.059), whereas *PARP10* knockout by guide RNA2 significantly impaired the proliferation of MOLM13 cells (*P* < 0.05) (Figure 6C and 6D). Thus, our bioinformatic analysis and functional experiments highlighted the important role of *PARP10* in AML cell proliferation, indicating that targeting *PARP10* might provide a novel strategy for treating AML.

## DISCUSSION

The prognosis of AML is poor due to its highly heterogeneous blast; thus, novel diagnostic and prognosis biomarkers are needed to better define the disease and improve stratified therapy [1, 3, 6]. The PARP family of proteins is composed of 17 members, which are responsible for adenosine diphosphate (ADP) ribosylation in the cells, while the PARP family has emerged as important regulatory factors in both DNA and cancer biology [8, 30]. PARP1/2 inhibitors were first approved for the treatment of breast and ovarian cancers [8, 13], and subsequently, many *in vitro* and *in vivo* studies have been conducted to investigate their efficacy in other tumor types, although without any satisfactory progress, which is partially due to mutations that affect the DDR-associated genes, which are not common in other malignancies, including AML [9, 12, 13, 15]. There is the possibility that AML survival is independent of PARP1/2, whereas the other PARP family proteins are essential in AML cells; thus, targeting the other PARP family proteins may provide promising cytotoxic activities against leukemia in the clinic. In this study, we comprehensively evaluated the differential expression pattern of PARP family genes across AML patients and healthy donors and analyzed the association between *PARP10* expression and clinical parameters as well as AML prognosis.

Through the integrated analyses of three large datasets encompassing gene expression data on AML samples and healthy controls (i.e., TCGA versus GTEX, GSE15061, and Bloodspot) [16–18], we found that the expressions of *PARP6* and *PARP10* were simultaneously higher in AML cells than in normal cells, while the differential expression patterns of the other genes showed discrepancies across the three datasets, which indicated that targeting *PARP6* and *PARP10* might offer the more plausible option than targeting PARP1/2 in precision medicine. To better understand the correlation between gene expression and the prognosis of AML, we performed survival analysis using the TCGA LAML dataset and found patients with higher expressions of *PARP10* correlated with inferior OS and EFS compared to patients with lower expressions of *PARP10*, whereas *PARP6* expression had no influence on the AML prognosis. Future *in vitro* and *in vivo* studies are warranted to dissect the role of *PARP10* on leukemia initiation and disease progression.

Survival analysis of the differentially expressed genes (i.e., *PARP6* and *PARP10*) suggested that *PARP10* was the only gene whose high expression was associated with inferior clinical outcomes. The Chi-square test of the *PARP10* expression group (i.e., high vs. low) and clinical characteristics demonstrated that the high expression of *PARP10* was associated with older age, more frequent *TP53* mutations, and higher risk classifications, all of which exhibited features of high AML risk and were consistent with the result of predicting inferior clinical outcomes. Intriguingly, multivariate Cox regression analysis identified *PARP10* as an independent factor in AML prognosis for both OS and EFS after adjusting for age (<60 vs. >60), WBC count (≥30 vs. <30 × 10^9^/L), *FLT3* and *TP53* statuses (wildtype vs. mutant), and transplant status (yes vs. no). All these results demonstrate that *PARP10* could be used as a potential biomarker, which might contribute to the precise prognosis and stratification of AML. More importantly, we found patients with higher expressions of *PARP10* would benefit from allo-SCT therapy, while patients with lower expressions of *PARP10* should receive allo-SCT due to the detection of an inferior EFS after this therapy according to our study. Therefore, it is important for clinical physicians or researchers to perform rt-qPCR or high-throughput RNA sequencing to quantify the expression value of *PARP10* in clinical management, based on which AML patients could be stratified into different risk group to receive comparable therapies. Furthermore, multicenter studies are warranted to validate these findings and to better stratify future AML therapies, and after that it should be considered that whether *PARP10* expression could be included into the novel ELN 2022 AML prognostic recommendations.

Previous studies demonstrated that *PARP10* functions as an oncogene in Hela cells [24], while acting as a tumor suppressor in hepatocellular carcinoma cells [31], indicating that it has a dual role in carcinogenesis, in a context-dependent manner. Herein, we analyzed the *PARP10* co-expression network in AML for the first time, and our results suggest that *PARP10* may work together with other signaling pathways to exert an oncogenic effect in AML, while it also provided hints regarding its underlying molecular mechanism in oncogenesis. Moreover, GSEA analysis indicated that high *PARP10* expression was involved in *MYC, NFKB*, and *EZH2* target gene sets in AML patients, providing a potential and interesting direction for further exploration of its biological functions. Intriguingly, *PARP10* knockout significantly impaired the proliferation of AML cells *in vitro*, potentiating that targeting *PARP10* might provide a novel strategy for the treatment of AML. Clinical trials evaluating PARP10 inhibition alone or in combination with other drugs in the treatment of AML are warranted to conduct in the future.

However, there are some limitations of the current study. The major limitation of public databases is the batch effect derived from high-throughput sequencing of large number of human samples. Bioinformatics researchers have been dedicated to developing novel algorithms to adjust batch effect, which is always not satisfactory enough when applied to human samples with such great heterogeneity. Another limitation of public databases is that the survival data is taken from clinical cohort decades ago, which might not be able to represent the current status as treatment for AML has been refined and long-term prognosis in patients with AML has gradually improved in recent decades. Thus, direct experiment in the lab (e.g., rt-qPCR) to compare the differential expression of *PARP10* in AML and normal cells, and association analysis of *PARP10* expression with clinical prognosis in the current treatment cohort are warranted to perform in the future.

In conclusion, our study revealed that *PARP10* was aberrantly expressed in AML patients, and its high expression was associated with high-risk factors and poor prognosis and could be an independent poor survival factor in AML patients. Importantly, our results suggested that patients with high expression of *PARP10* would benefit from allo-SCT therapy, while those with low expression of *PARP10* should not receive allo-SCT due to an inferior EFS after allo-SCT use.

## Supplementary Materials

Supplementary Figures

Supplementary Tables 1-3

Supplementary Table 4
