# Supplementary material for: PARP10 is highly expressed and associated with inferior outcomes in acute myeloid leukemia
Source: Aging (Albany NY). 2023 Jul 27;15(14):6757–73. doi: 10.18632/aging.204832 (PMC10415541; doi:10.18632/aging.204832)
Supplement: Supplementary Figures [file aging-15-204832-s001.pdf]

SUPPLEMENTARY FIGURES

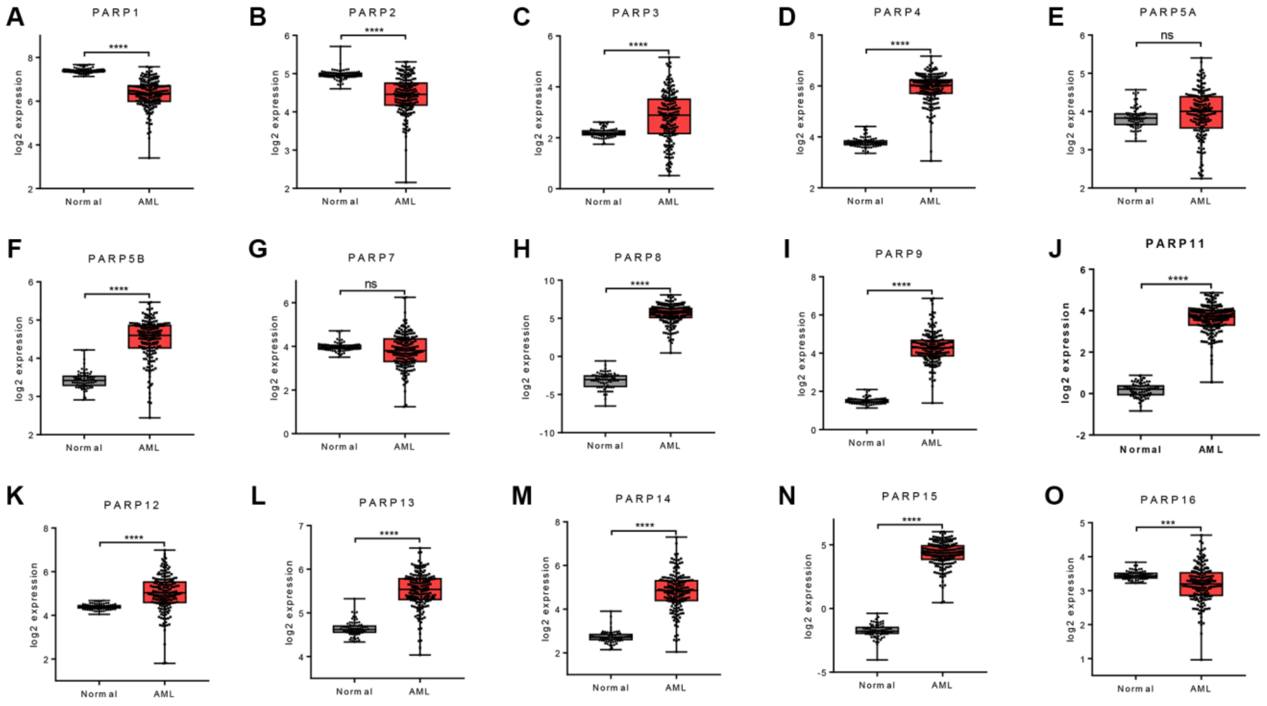

Supplementary Figure 1. Expression differences of *PARPs* between AML samples and normal controls in TCGA versus GTEX datasets. (**A**) *PARP1*, (**B**) *PARP2*, (**C**) *PARP3*, (**D**) *PARP4*, (**E**) *PARP5A*, (**F**) *PARP5B*, (**G**) *PARP7*, (**H**) *PARP8*, (**I**) *PARP9*, (**J**) *PARP11*, (**K**) *PARP12*, (**L**) *PARP13*, (**M**) *PARP14*, (**N**) *PARP15* and (**O**) *PARP16.* Unpaired *t* test was used to estimate the significance of expression difference. $^{*}P < 0.05$; $^{**}P < 0.01$; $^{***}P < 0.005$; $^{****}P < 0.001$. Abbreviation: ns: not significant.

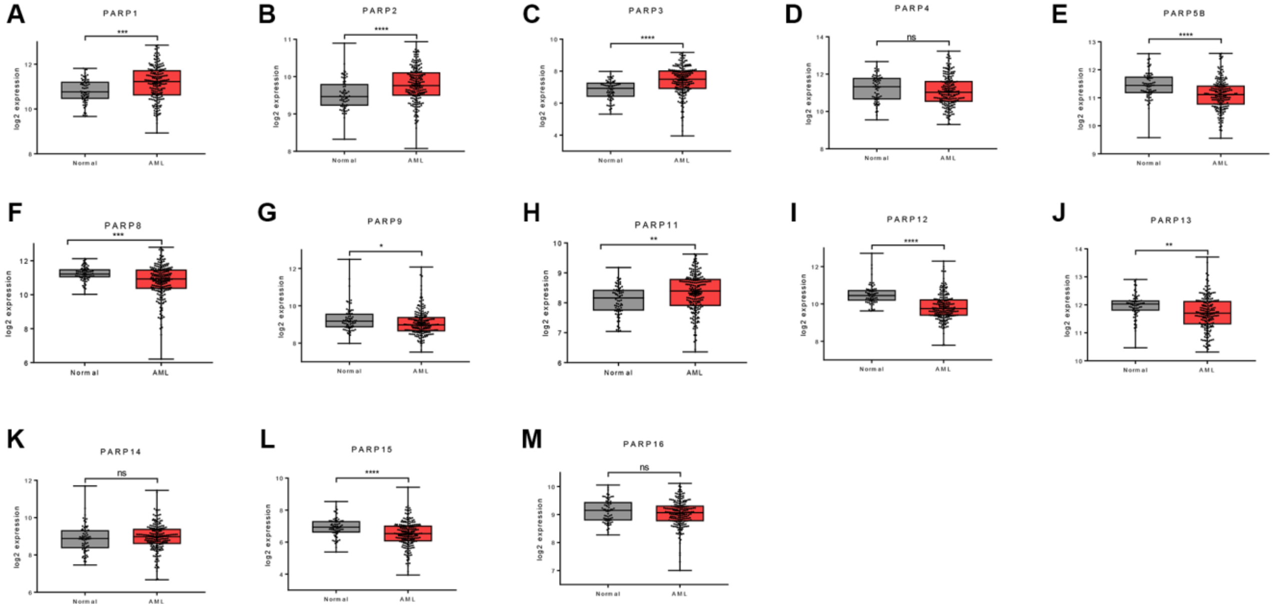

Supplementary Figure 2. Expression differences of *PARPs* between AML samples and normal controls in GSE15061 datasets. (**A**) *PARP1*, (**B**) *PARP2*, (**C**) *PARP3*, (**D**) *PARP4*, (**E**) *PARP5B*, (**F**) *PARP8*, (**G**) *PARP9*, (**H**) *PARP11*, (**I**) *PARP12*, (**J**) *PARP13*, (**K**) *PARP14*, (**L**) *PARP15* and (**M**) *PARP16.* Unpaired *t* test was used to estimate the significance of expression difference. $^{*}P < 0.05$; $^{**}P < 0.01$; $^{***}P < 0.005$; $^{****}P < 0.001$. Abbreviation: ns: not significant.

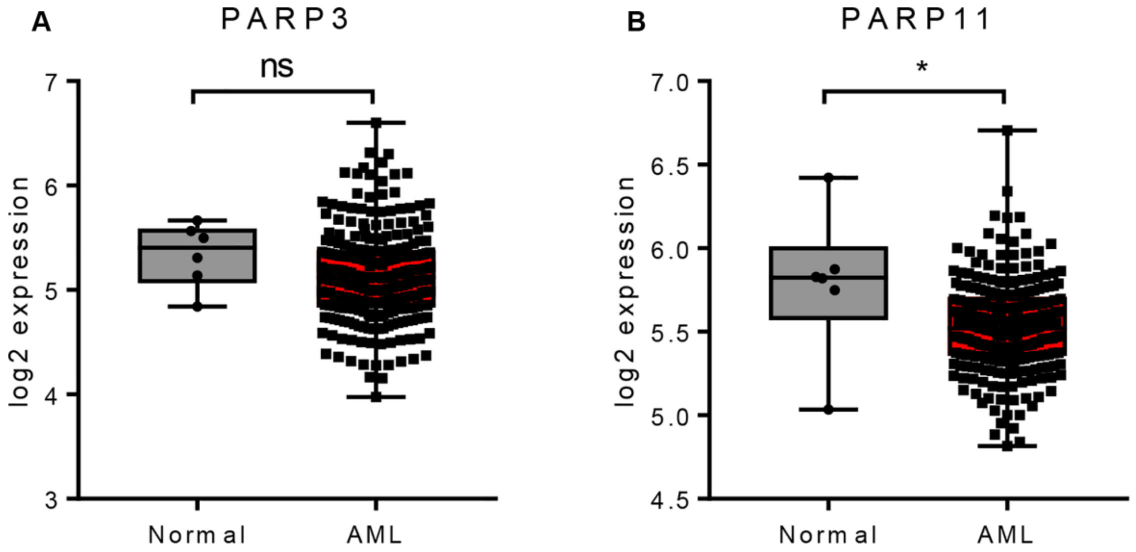

Supplementary Figure 3. Expression differences of *PARPs* between AML samples and normal controls in Bloodspot datasets. (**A**) *PARP3* and (**B**) *PARP11*. Unpaired *t* test was used to estimate the significance of expression difference. $^{*}P < 0.05$. Abbreviation: ns: not significant.

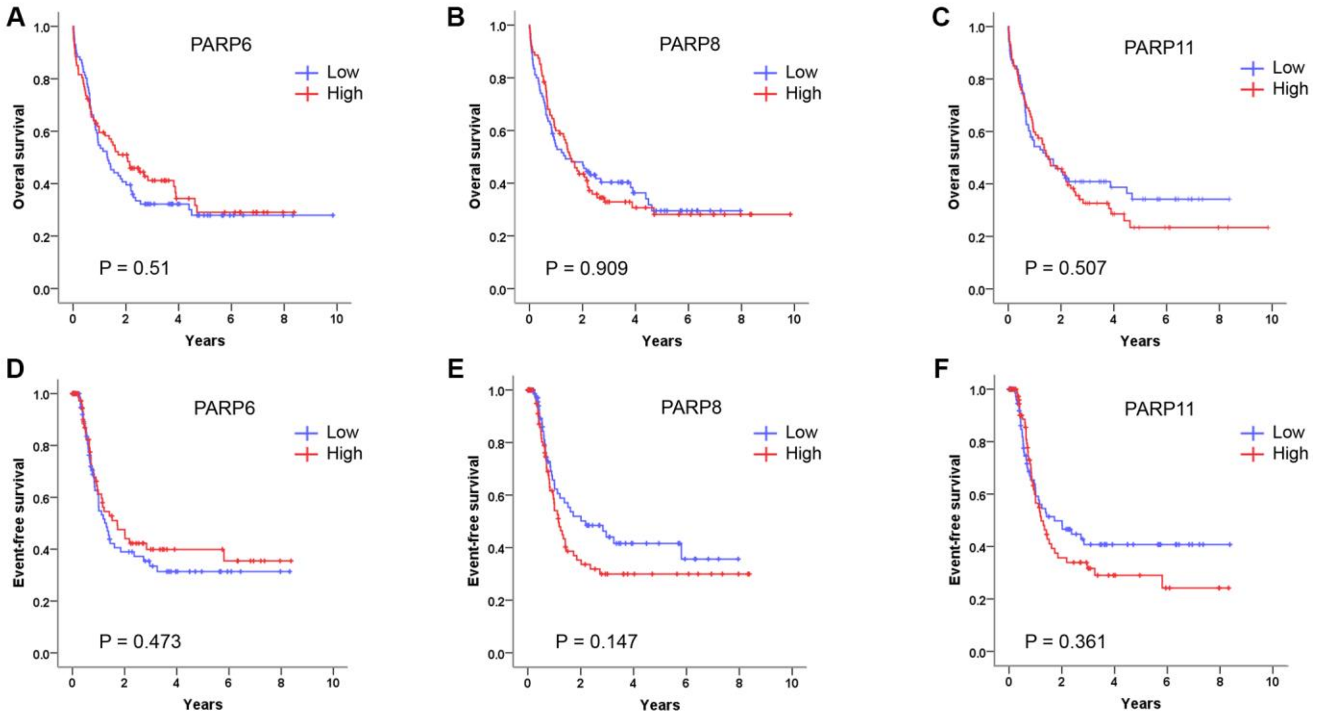

Supplementary Figure 4. Survival analysis of AML patients according to *PARPs* expression. Overall survival rate of AML patients with high expression versus low expression of *PARPs*, (**A**) *PARP6*, (**B**) PARP8 and (**C**) *PARP11*. Event-free survival rate of AML patients with high expression versus low expression of *PARPs*, (**D**) *PARP6*, (**E**) *PARP8* and (**F**) *PARP11*. Log-rank test was used to generate the survival curves and analyze the survival difference between the high and the low expression groups.

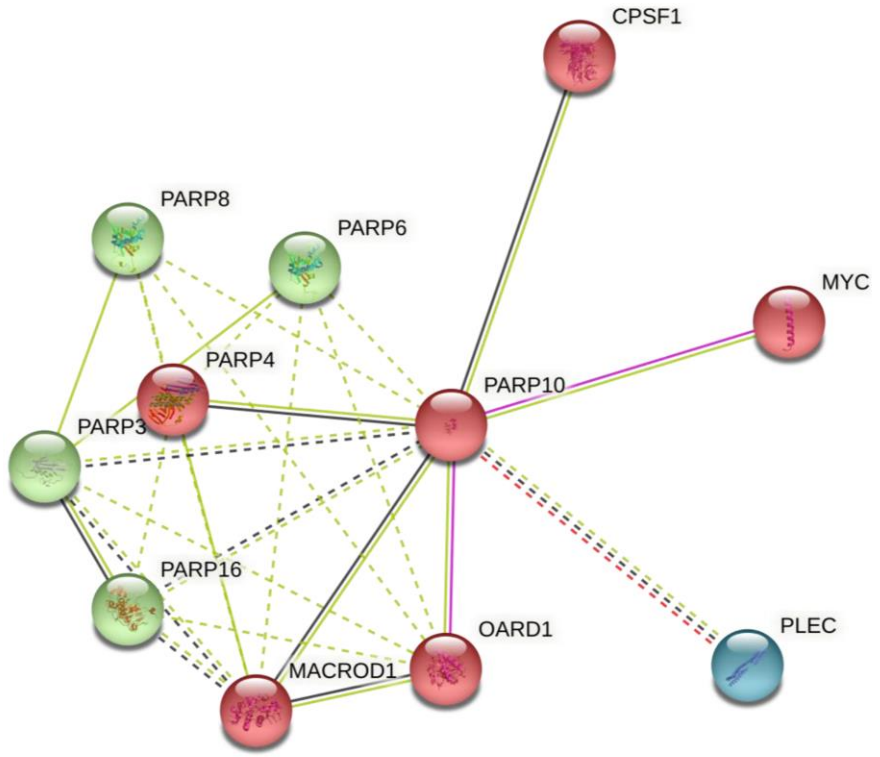

Supplementary Figure 5. STRING protein–protein interaction network analysis of *PARP10.*
