# Supplementary material for: PARP10 is highly expressed and associated with inferior outcomes in acute myeloid leukemia
Source: Aging (Albany NY). 2023 Jul 27;15(14):6757–73. doi: 10.18632/aging.204832 (PMC10415541; doi:10.18632/aging.204832)
Supplement: Supplementary Table 4 [file aging-15-204832-s003.pdf]

SUPPLEMENTARY TABLE

Supplementary Table 4. Oligo sequences of *PARP10* guide RNAs and genotyping primers.

**1. Oligo sequences for *PARP10* guide RNAs**	
sgRNA1-F	caccGCAGCTCGTCGGGCACGGCA
sgRNA1-R	AAACTGCCGTGCCCGACGAGCTGC
sgRNA2-F	caccgTTTGAAAACCGCCGACGCTC
sgRNA2-R	AAACGAGCGTCGGCGGTTTTCAAAc
**2. Genotyping primers for validation of *PARP10* editing mediated by CRISPR/Cas9**	
genotyping-F	GCAGGATGTCAGGCATTAGAATA
genotyping-R	GGGGAGCATTGAGGACACACCTTG
